# Prevalence of Tricuspid Regurgitation and Mortality Outcomes in Patients With Heart Failure: Stratification by Phenotype

**DOI:** 10.1016/j.jacadv.2026.102612

**Published:** 2026-02-18

**Authors:** Isabel Mattig, Elena Romero Dorta, David Frumkin, Hannes Kretzschmar, Alexander Sommer, Alva Morgane Redel, Johannes Buchstaller, Karl Stangl, Gerhard Hindricks, Henryk Dreger

**Affiliations:** a Deutsches Herzzentrum der Charité, Department of Cardiology, Angiology and Intensive Care Medicine, Berlin, Germany; b Charité, Universitätsmedizin Berlin, Corporate Member of Freie Universität Berlin and Humboldt-Universität zu Berlin, Berlin, Germany; c DZHK (German Centre for Cardiovascular Research), Partner Site Berlin, Germany; d Berlin Institute of Health at Charité – Universitätsmedizin Berlin, BIH Biomedical Innovation Academy, Berlin, Germany; e Deutsches Herzzentrum der Charité, Department of Cardiology, Angiology and Intensive Care Medicine, Campus Virchow Klinikum, Berlin, Germany; f Structural Heart Interventions Program (SHIP), Deutsches Herzzentrum der Charité, Berlin, Germany

**Keywords:** heart failure, prevalence, prognosis, tricuspid regurgitation

**What is the clinical question being addressed?**

What is the natural course of different tricuspid regurgitation (TR) grades in all heart failure phenotypes?

**What is the main finding?**

TR prevalence, severity, and the 2-year mortality risk in patients with at least moderate TR are comparable across heart failure subgroups.

Significant tricuspid regurgitation (TR), the most common secondary functional TR, is a frequent finding associated with substantial mortality.^1^ Recent studies focused on TR in mixed heart failure (HF) populations or subgroups, but did not compare heart failure with reduced ejection fraction (HFrEF), heart failure with mildly reduced ejection fraction (HFmrEF), and heart failure with preserved ejection fraction (HFpEF).^1^^,^^2^ Since novel interventional therapies have predominantly been used in HFpEF, a comprehensive understanding of TR across all HF phenotypes is crucial to identify patients at risk for TR-related adverse events. The present study investigated the prevalence and all-cause mortality in patients with different TR grades and HF phenotypes.

## Methods

The study design was described previously.^3^ Briefly, from 2020 to 2022, we prospectively enrolled adults with HFrEF (left ventricular ejection fraction [LVEF] ≤40%), HFmrEF (LVEF 41%-49%), or HFpEF (LVEF ≥50%) who were admitted to the departments of cardiology at 2 sites of our institution (NCT04570098).^3^ TR was assessed at the initial visit or within a maximum of 3 months before enrollment. Moderate and severe TR were comprehensively quantified by measuring the effective regurgitant orifice area and regurgitant volume using the proximal isovelocity surface area, vena contracta, and hepatic vein reflux following volume optimization. Follow-up telephone calls were performed at 3, 6, 12, and 24 months.^3^ The primary outcome was all-cause mortality. The study was authorized by the Institutional Review Board of the Charité–Universitätsmedizin Berlin (EA1/178/19).^3^ All patients provided written informed consent.

Statistical analysis was conducted with SPSS Statistics (version 28) for Windows (IBM Corporation) and R environment (4.3.3, R Foundation for Statistical Computing). Data are presented as mean ± SD, median with 25th-75th percentiles (Q1-Q3) or count (percentage). Kaplan-Meier curves and Cox regression adjusted for age, sex, coronary artery disease, cardiac implantable electronic device, and arterial hypertension were used to compare mortality across HF groups and are presented HRs with 95% CIs.

## Results

After screening 1,394 patients with HF, we enrolled 1,012 HF patients, including 336 subjects with HFrEF (33.2%), 195 with HFmrEF (19.3%), and 481 with HFpEF (47.5%). Overall, 382 patients were excluded due to language barriers (n = 166), lack of a permanent residence or a telephone (n = 6), or because they declined participation (n = 210). In the overall study cohort, 628 patients (62.1%) were males, the median age was 77 years (Q1-Q3: 68-82 years), and 525 patients (51.9%) suffered from NYHA functional class III. Twenty-seven percent of patients were admitted due to decompensated HF and 51.0% received an intervention such as percutaneous coronary intervention or catheter ablation. The most common comorbidities were arterial hypertension (75.0%), coronary artery disease (56.1%), and atrial fibrillation or flutter (55.5%). Twenty percent of HF patients suffered from severe aortic stenosis and 13.0% from severe mitral regurgitation. N-terminal pro–B-type natriuretic peptide values reached from 1,393 ng/L (Q1-Q3: 628-3,028 ng/L) in HFpEF to 3,443 ng/L (Q1-Q3: 1,401-10,005 ng/L) in HFrEF patients. The mean systolic pulmonary artery pressure was 42 ± 16 mm Hg without significant differences between the phenotypes. Right ventricular function measured by tricuspid annular plane systolic excursion was 17 ± 5 mm in HFrEF, 19 ± 5 mm in HFmrEF, and 21 ± 5 mm in HFpEF patients. The majority of patients received HF therapy, including 692 patients (68.4%) on diuretic agents, 691 (68.3%) on beta-blockers, 758 (74.9%) on angiotensin-converting enzyme or angiotensin receptor-neprilysin inhibitors, and 413 (40.8%) on mineralocorticoid receptor antagonists at discharge. Sodium-glucose transport protein 2 inhibitors were given to 206 HF patients (20.4%). Diuretic agents, mineralocorticoid receptor antagonists, and sodium-glucose transport protein 2 inhibitors were more commonly prescribed in HFrEF compared to HFmrEF and HFpEF.

**Figure 1:**
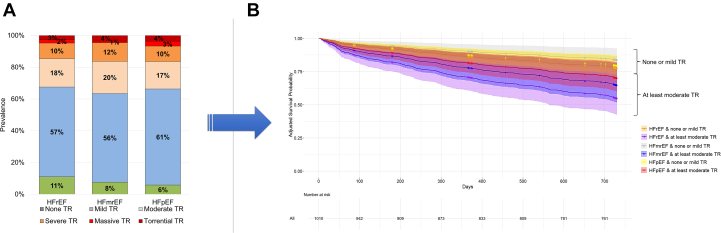
**Prevalence and Prognostic Significance of Tricuspid Regurgitation Across Different HF Phenotypes** (A) A total of 1,012 HF patients with a transthoracic echocardiography were prospectively enrolled, with 33% of patients with HFrEF, 19% with HFmrEF, and 48% with HFpEF. (B) Adjusted 2-year survival was similar across HF phenotypes within both TR severity groups (none/mild and at least moderate). HF = heart failure; HFrEF = heart failure with reduced ejection fraction; HFmrEF = heart failure with mildly reduced ejection fraction; HFpEF = heart failure with preserved ejection fraction; TR = tricuspid regurgitation.

In the overall HF cohort, 160 patients (15.9%) had severe TR, 182 (18.0%) moderate, 590 (58.3%) mild TR, and 80 patients (7.9%) no or trace TR. The distribution of TR in different HF phenotypes was comparable (Figure 1A). TR etiology included mixed pathology (n = 126, 37.4%), atrial (n = 113, 33.3%), cardiac implantable electronic device–related (n = 36, 10.8%), and ventricular TR (n = 36, 10.5%) without significant differences between the HF entities. After a median of 728 days (Q1-Q3: 709-730 days), 1,010 (99.8%) patients had at least 1 follow-up to assess the survival status. All-cause mortality was 24.9% (n = 244) including 10.1% cardiac and 13.2% noncardiac cause of death (unknown cause in 1.6%). After adjustment, there was an increased mortality risk in patients with at least moderate TR compared to none or mild in all HF subgroups (HFrEF HR: 2.25 [95% CI: 1.46-3.48], HFmrEF HR: 2.58 [95% CI: 1.32-5.04], and HFpEF HR: 1.65 [95% CI: 1.12-2.44]). Across HF subgroups, mortality risk did not differ among patients with none or mild TR (HFrEF reference category, HFmrEF HR: 0.64 [95% CI: 0.37-1.11], HFpEF HR: 0.78 [95% CI: 0.51-1.19]), nor among those with at least moderate TR (HFrEF reference category, HFmrEF HR: 0.77 [95% CI: 0.46-1.29], HFpEF HR: 0.67 [95% CI: 0.43-1.05]) (Figure 1B).

In summary, our main findings are: 1) The prevalence of at least moderate TR was substantial across all three HF phenotypes, affecting ∼1 in 3 patients, and associated with an increased risk for all-cause mortality; 2) the distribution of TR severity did not differ significantly among the HF subgroups; and 3) adjusted 2-year survival probability did not differ between HF phenotypes, irrespective of whether patients had none/mild TR or at least moderate TR.

## Discussion

Comparable data were reported by Heitzinger et al,^4^ who retrospectively analyzed a cohort of HF patients but focused solely on secondary TR and excluded those with other valvular defects. A significantly higher prevalence of severe TR was observed in HFrEF (20%) relative to other HF subgroups.^4^ In contrast, our data revealed comparable TR severity distribution among HF phenotypes, suggesting a more uniform burden of TR. In agreement with our results, various studies reported increased mortality with increasing TR severity.^2^^,^^4^

### Study limitations

The study is subject to referral bias as it draws on data from 2 sites of a single university hospital. Follow-up relied primarily on patient telephone interviews, which may introduce reporting bias; additional information from relatives, physicians, and official sources completed missing data. Transthoracic echocardiograms were interpreted locally by 2 to 3 experienced cardiologists rather than by a core laboratory. The multivariable cox regression model included demographic variables and comorbidities that differed across HF phenotypes. However, the adjustment was constrained by the limited number of events in smaller subgroups and thus, not all variables that may affect mortality could be considered (eg, right ventricular function, severe aortic stenosis, and severe mitral regurgitation).

## Conclusions

Significant TR is common across all HF phenotypes and is consistently associated with worse outcomes. Our prospective study shows that TR prevalence and severity are similar in HFpEF, HFmrEF, and HFrEF, and that mortality risk within each TR severity category does not differ by HF phenotype. Patients with at least moderate TR had markedly poorer survival, underscoring the need for early recognition and proactive management across the HF spectrum.

## Funding support and author disclosures

The study was funded by 10.13039/100000046Abbott, Chicago, Illinois, USA. Dr Mattig is a participant in the BIH Charité Clinician Scientist Program funded by the Charité–Universitätsmedizin Berlin and the Berlin Institute of Health at Charité (BIH). Dr Dregen has received speaker fees and research support from Abbott and Edwards Lifesciences. All other authors have reported that they have no relationships relevant to the contents of this paper to disclose.
